# CAVEOLIN-1 expression in brain metastasis from lung cancer predicts worse outcome and radioresistance, irrespective of tumor histotype

**DOI:** 10.18632/oncotarget.4988

**Published:** 2015-07-22

**Authors:** Eleonora Duregon, Rebecca Senetta, Alessandra Pittaro, Ludovica Verdun di Cantogno, Giulia Stella, Pierpaolo De Blasi, Michele Zorzetto, Cristina Mantovani, Mauro Papotti, Paola Cassoni

**Affiliations:** ^1^ Department of Oncology, University of Torino at San Luigi Hospital, Orbassano, Turin, Italy; ^2^ Laboratory of Biochemistry and Genetics, Pneumology Unit, Department of Molecular Medicine University and Fondazione IRCCS Policlinico San Matteo, Pavia, Italy; ^3^ University of Torino and Collegio Carlo Alberto, Torino, Italy; ^4^ Department of Oncology, Radiation Oncology, University of Torino, Italy; ^5^ Department of Medical Sciences, University of Torino, Italy

**Keywords:** Caveolin 1, brain metastasis, non-small-cell lung cancer, radiotherapy

## Abstract

Brain metastases develop in one-third of patients with non-small-cell lung cancer and are associated with a dismal prognosis, irrespective of surgery or chemo-radiotherapy. Pathological markers for predicting outcomes after surgical resection and radiotherapy responsiveness are still lacking. Caveolin 1 has been associated with chemo- and radioresistance in various tumors, including non-small-cell lung cancer. Here, caveolin 1 expression was assessed in a series of 69 brain metastases from non-small-cell lung cancer and matched primary tumors to determine its role in predicting survival and radiotherapy responsiveness. Only caveolin 1 expression in brain metastasis was associated with poor prognosis and an increased risk of death (log rank test, *p* = 0.015). Moreover, in the younger patients (median age of <54 years), caveolin 1 expression neutralized the favorable effect of young age on survival compared with the older patients. Among the radiotherapy-treated patients, an increased risk of death was detected in the group with caveolin 1-positive brain metastasis (14 out of 22 patients, HR=6.839, 95% CI 1.849 to 25.301, Wald test *p* = 0.004). Overall, caveolin 1 expression in brain metastasis from non-small-cell lung cancer is independently predictive of worse outcome and radioresistance and could become an additional tool for personalized therapy in the critical subset of brain-metastatic non-small-cell lung cancer patients.

## INTRODUCTION

Brain metastasis from non-small-cell lung cancer (NSCLC), the leading cause of cancer-related death worldwide, develops in 20-40% of patients [1, 2] at a relatively earlier stage compared with other primary cancers. It is associated with a dismal prognosis, with a median survival of one month in untreated patients. Other than supportive care with corticosteroids, surgery, whole-brain radiation therapy (WBRT), stereotactic radiosurgery and chemotherapy, either alone or in various combinations, are valuable options for these patients [3]. Although WBRT is still the most effective treatment following surgery [3], the overall survival is about five months after WBRT, and only 5% of patients survive one year [4, 5]. Prognostic factors affecting survival include age, performance status, the number, locations, and sizes of brain metastases, the presence or absence of symptoms and/or mass effect, suitability for surgical resection, and availability (or not) of options for controlling extracranial disease [6]. However, pathological tools for selecting patients who may benefit from surgical resection of brain metastasis, for predicting outcomes after surgical resection and for predicting radiotherapy responsiveness are lacking.

Among various prognostic and predictive markers, caveolin 1 (Cav1) has been associated with chemoresistance in various solid tumors, including NSCLC [7, 8, 9], and with radioresistance in pancreatic [10, 11] and colon cancers [12]. Cav1 is an essential structural constituent of caveolae, acting as a multifunctional scaffolding protein with multiple binding partners. Its ability to interact with numerous proteins makes it a central cellular signaling molecule. Cav1 organizes signaling complexes at the inner plasma membrane to activate a variety of cellular events and has been implicated in neurodegenerative diseases, diabetes mellitus, senescence and oncogenesis [13]. In cancer, Cav1 is involved in cellular transformation, tumor growth, cell death and survival, multidrug resistance, angiogenesis, cell migration and metastasis [14]. A biphasic role of Cav1 in signal transduction and cancer has been thoroughly documented. It acts as a tumor-suppressing protein during the early stages of cancer progression, but once it becomes upregulated during more advanced stages of disease, it gains an oncogenic function, which contributes to an aggressive and metastatic phenotype [14]. In agreement with this oncogenic potential, *in vitro* Cav1 knockdown of NSCLC cells has been shown to result in inhibition of cellular proliferation [15], whereas Cav1-overexpressing NSCLC cells, as well as anoikis-resistant cells, exhibit enhanced metastatic activity [16]. Meta-analysis of NSCLC patients [17] has revealed that although the Cav1 level is significantly lower in cancerous tissues than in non-neoplastic lung tissues, Cav1-expressing NSCLC patients have a higher risk of death and reduced progression-free survival, consistent with a role of Cav1 in tumor progression. We have previously described the differential expression of Cav1 according to NSCLC histotype and its increased expression in brain metastasis [18].

To the best of our knowledge, neither a hypothetical prognostic role of Cav1 expression in brain metastasis from NSCLC nor its relationship with response to radiotherapy has been assessed. Therefore, we evaluated the role of Cav1 in predicting survival and radiotherapy responsiveness in 69 patients with NSCLC with brain metastasis. We found that a) Cav1 expression in brain metastasis was related to poor prognosis; b) in younger patients, Cav1 expression in brain metastasis resulted in an increased risk of and neutralization of the favorable impact of young age on survival; and c) Cav1 positivity in brain metastasis was predictive of radioresistance.

## RESULTS

### Clinico-pathological data and Cav1 protein expression

**Figure 1:**
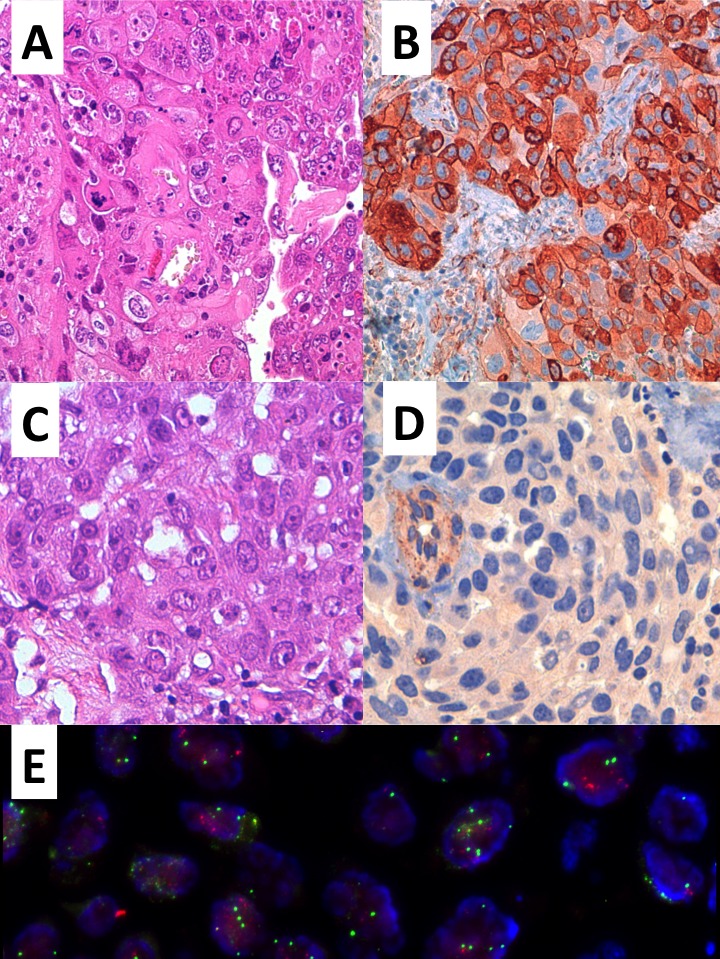
Representative examples of two brain metastasis from lung adenocarcinoma (A, C hematoxylin & Eosin, original magnification 400X) with mild to strong membrane-cytoplasmic immunoreactivity and negative staining for Cav1 (respectively B, D original magnification 400X) and high CAV1 gene polysomy by FISH analysis (E)

The main clinical and pathological features of the whole series of 69 cases are summarized in Table 1. A total of 38 cases (55%) were adenocarcinomas. Cav1 expression in primary lung carcinoma and matched brain metastasis exhibited a significant association (chi-square test *p* < 0.001). Overall, 16 (23%) primary tumors and 28 brain metastases (41%) expressed Cav1, with mild to strong membrane or cytoplasmic staining of neoplastic cells (Figure 1). Fourteen negative primary tumors shifted to Cav1 positivity during metastasis, whereas two positive lung carcinomas lost Cav1 expression in the brain metastatic tissue (Table 2). Cav1 brain metastasis expression and histotype were significantly associated (chi-square test *p* = 0.043) because all of the brain neuroendocrine tumors (7 cases) were negative for Cav1 expression. Analysis of the differential expression of Cav1 by histotype revealed that all of the positive primary adenocarcinomas (7 cases) maintained Cav1 expression at the brain metastatic site and that 7 out of 31 acquired Cav1 expression during metastasis (chi-square test *p* < 0.001) (Table 3). No association of Cav1 expression was found for squamous cell or large cell carcinoma histotype (Table 4).

**Table 1:** Clinical and pathological features of 69 patients with NSCLC metastatic to the brain analyzed for Cav1 expression

Parameter	
**M/F ratio**	3.31
**Age, mean (years) [range]**	64 [35-80]
**Number of brain metastases**	single: 49 multiple: 20
**Histotype**	adenocarcinoma: 38 squamous cell carcinoma: 14 neuroendocrine carcinoma: 7 large cell carcinoma: 10
**Type of radiotherapy**	adjuvant : 22 cytoreductive: 9
**Disease status** *(lost to FU: 1)*	NED/DOC: 14 DOD: 57
**Median overall survival (months)**	14 [1-84]

**Table 2:** Distribution of Cav1 positivity in primary tumors and brain metastases

Primary tumor	Metastasis		Sum	*p*
	Cav1 negative	Cav1 positive		
**Cav1 negative**	39	14	53	*p* < 0.001
**Cav1 positive**	2	14	16	
**Sum**	41	28	69	

**Table 3:** Differential expression of Cav1 by histotype and site

	Primary lung cancer		*p*	Brain metastasis		*p*
	Cav1 negative	Cav1 positive		Cav1 negative	Cav1 positive	
**Adenocarcinoma** **Squamous cell** **Neuroendocrine** **Large cell**	31 9 7 6	7 5 0 4	0.143	24 6 7 4	14 8 0 6	0.043

**Table 4:** Correlation between Cav1 expression at primary and metastatic sites in adenocarcinoma histotype

	Primary tumor adenocarcinoma	Metastasis		*p*
		Cav1 negative	Cav1 positive	
**Adenocarcinoma**	**Cav1 negative**	24	7	*p* < 0.001
	**Cav1 positive**	0	7	
**Squamous cell**	**Cav1 negative** **Cav1 positive**	4 2	5 3	1
**Neuroendocrine**	**Cav1 negative** **Cav1 positive**	7 0	0 0	not available
**Large cell**	**Cav1 negative** **Cav1 positive**	4 0	2 4	0.1472

### Cav1 mutation and FISH analyses

Fifty-four cases were suitable for mutational analysis. The *Cav1* P132L mutation was found in two lung adenocarcinomas, only one of which retained the mutation at the metastatic site. Interestingly, this case had a negative immunohistochemistry result, whereas the other case showed positive results at the primary and secondary sites. FISH analysis was performed on 16 matched cases, among which six displayed a gain and three exhibited amplification of Cav1 expression at both the primary and metastatic sites. The presence of a gain or amplification of expression in the primary neoplasm was significantly associated with the same event in the metastasis (Fisher's exact test *p* = 0.01). None of the cases acquired a gain or amplification in the metastatic lesion. The two mutated cases showed neither a gain nor amplification.

### Survival analysis of Cav1 expression and responsiveness to radiotherapy

**Figure 2:**
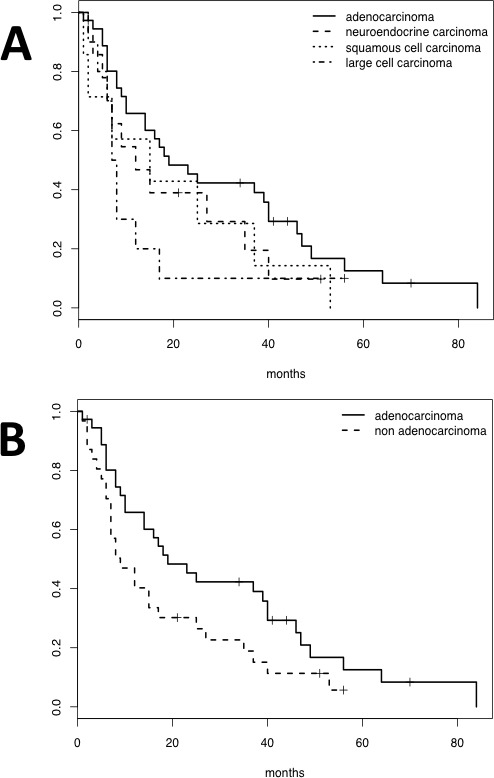
Survival curves showing that grouping by histotype was not a significant predictor for survival (Log rank test, p=0.233, 2A) Conversely, adenocarcinoma was associated with prolonged survival when other histotypes were considered together (Log rank test, p=0.056, 2B).

**Figure 3:**
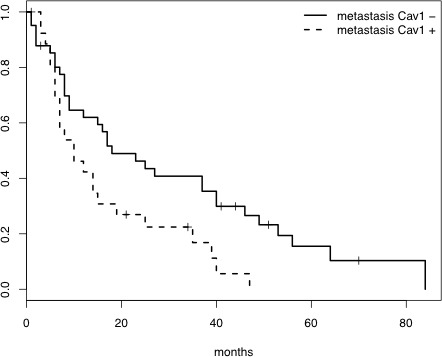
Survival curves demonstrating the association between Cav1 expression in brain metastasis and poor prognosis (Log rank test, p=0.015)

Follow-up data, ranging from 1 to 84 months (median overall survival: 14 months), were available for all but one patient. At the time of analysis, 57 patients (84%) had died, all because of brain metastasis. Among the 11 (16%) who were living, two were Cav1 positive in both the primary lung lesion and brain metastasis, whereas one had acquired Cav1 expression in the metastasis. Overall, although grouping by histotype did not reveal differences in survival (log rank test, *p* = 0.233, Figure 2A), the adenocarcinoma histotype was associated with prolonged survival (log rank test, *p* = 0.056, median survival of 19 *vs.* 9 months for cases with adenocarcinoma and with other histotypes considered together, respectively, Figure 2B). Only Cav1 expression in the brain metastasis (and not its expression in the primary lesion) was related to poor prognosis (log rank test, *p* = 0.015, median survival of 18 *vs.* 10 months for Cav1 negative and positive cases, respectively) (Figure 3). The presence of multiple metastases *vs.* a single brain metastasis (20 and 49 cases, respectively) was not associated with a significant difference in survival (log rank test, *p* = 0.999). With a proportional hazard model controlling for age at diagnosis (HR = 1.061, 95% CI 1.022 to 1.101, Wald test *p* = 0.002) and the adenocarcinoma histotype (HR = 0.521, 95% CI 0.301 to 0.901, Wald test *p* = 0.020), Cav1 expression in the metastasis (HR = 2.359, 95% CI 1.309 to 4.251, Wald test *p* = 0.004) was associated with an increased risk of death, demonstrating its value as an independent predictor (Table 5a). To study the interaction between age and Cav1 expression at the metastatic site, we considered age as a factor, establishing “young” (age below the median of 63) and “old” (age ≥ 64) classifications. Interestingly, Cav1-positive metastasis was associated with an equivalent increase in the risk of death in the two age groups (interaction term, Wald test *p* = 0.780) and neutralized the favorable effect of young age on survival compared with the older patients (old Cav1 negative *vs.* young Cav1 positive, HR = 1.058, 95% CI 0.537 to 2.083, Wald test *p* = 0.870) (Table 5b).

**Table 5:** Proportional hazard model for the whole data set

	Without interaction (a)		With interaction (b)	
	HR	*p*-value	HR	*p*-value
**Age**	1.061	***0.002***	--	--
**Old age**	--	--	2.430	***0.026***
**Histotype**	0.521	***0.020***	0.522	***0.021***
**Cav1 positive metastasis**	2.359	***0.004***	2.571	***0.031***
**Interaction effect Cav1/age**	--	--	1.183	***0.780***

Evaluation of the patients with available brain radiotherapy data (49 patients, 44 deaths), controlling for age at diagnosis, the adenocarcinoma histotype and Cav1 expression in the brain metastasis, revealed that radiotherapy was not significantly associated with survival (Table 6a). However, when accounting for an interaction between Cav1 expression and radiotherapy, the treatment was associated with a reduced risk of death in the patients with Cav1-negative brain metastasis (17 out of 27 cases, HR = 0.411, 95% CI 0.175 to 0.966, Wald test *p* = 0.041) and with an increased risk of death in those with Cav1-positive brain metastasis (14 out of 22 cases, HR = 6.839, 95% CI 1.849 to 25.301, Wald test *p* = 0.004) (Table 6b). In this subgroup of radiotherapy-treated patients, whole-brain radiotherapy was administered with adjuvant intent to 22 patients with a single surgically removed lesion and with palliative-cytoreductive intent to the remaining 9 patients with multiple brain lesions. Table 7 reports an extension of analysis using the proportional hazard model, accounting for radiotherapy intent (adjuvant or cytoreductive). The hazard ratios were found to be similar for the adjuvant and cytoreductive intents (HR = 0.383 *vs*. HR = 0.475, respectively) and likewise for the two interaction terms (Cav1/adjuvant HR = 7.17 *vs*. Cav1/cytoreductive HR = 6.534). Model selection based on log-likelihood ratio tests (*p*-value = 0.934) favored the model with interactions that did not account for the radiotherapy intent (Table 6b).

**Table 6:** Proportional hazard models restricted to radio-treated patients

	Without interaction (a)		With interaction (b)	
	HR	*p*-value	HR	*p*-value
**Age**	1.059	0.018	1.069	0.008
**Histotype adenocarcinoma**	0.388	0.004	0.321	<0.001
**Cav1-positive metastasis**	3.197	0.003	1.197	0.720
**Radiotherapy**	1.034	0.920	0.411	0.041
**Interaction effect Cav1/Radiotherapy**	--	--	6.839	0.004

**Table 7:** Proportional hazard model differentiating for adjuvant or palliative-cytoreductive radiotherapy treatment

	HR	*p*-value
**Age**	1.067	***0.014***
**Histotype adenocarcinoma**	0.312	***<0.001***
**Cav1-positive metastasis**	1.180	0.740
**Adjuvant Radiotherapy**	0.383	***0.050***
**Palliative-cytoreductive Radiotherapy**	0.475	0.210
**Interaction effect Cav1/ Adjuvant Radiotherapy**	7.177	***0.007***
**Interaction effect Cav1/ Cytoreductive Radiotherapy**	6.534	***0.043***

## DISCUSSION

In this study, we found that Cav1 expression in resected brain metastasis from NSCLC is a reliable predictor of poor survival and an independent predictor of poor radiotherapy responsiveness.

Prognostic role of adenocarcinoma histotype in brain metastasis: Lung cancer is the most frequent site of origin for brain metastasis, which is diagnosed in approximately 20-30% of patients during the course of their disease [2]. Approximately half of patients have a single brain metastasis [20]. Brain metastasis is more frequently found in NSCLC patients [21, 22], and these patients are usually considered terminal and to have poor prognosis. According to current guidelines [23, 24], resection of solitary brain metastasis is a valuable treatment option, resulting in a five-year survival rate of 20-25% if radical surgery of the primary tumor is also performed [25]. In agreement with a recent systematic review [26], survival improves when both the primary lung tumor and brain metastasis are resected; thus, removal of the primary site and single brain metastasis appears to be an effective treatment for long-term survival. However, effective tools for selecting patients who may benefit from surgical resection of brain metastasis remain to be identified. With respect to systemic therapy, tumor features, such as the histological subtype and molecular signature, play relevant prognostic and predictive roles because different NSCLC histologies have different treatment approaches and prognoses [27]. Surgical resection of brain metastasis from lung squamous cell carcinoma results in a significantly worse survival rate compared with other histotypes, whereas patients with adenocarcinoma live significantly longer [28]. Consistent with these findings, we found that the adenocarcinoma histotype is associated with prolonged survival, and advanced age is linked with poor prognosis.

Prognostic role of Cav1 expression in brain metastasis: Expression of Cav1 in human cancer cell lines and tumor samples has been documented in numerous studies, and it is increasingly clear that its role depends on the tumor type and stage [29]. In advanced stages, Cav1 gains an oncogenic role because its increased level favors the development of cellular traits associated with enhanced malignancy, including multidrug resistance and metastasis [30]. Accordingly, high Cav1 expression is a negative prognostic factor for overall and disease-free survival in patients with various tumor types, including breast [31], esophagus [32], pancreas [33], kidney [34], and prostate tumors [35], meningioma [36] and oligodendroglioma [37]. Concerning lung cancer, normal human lung epithelial cultures abundantly express Cav1, whereas its expression is reduced or absent in 95% of small-cell lung cancer cell lines and is retained in 76% of NSCLC cells [15]. In NSCLC cell lines, Cav1 expression increases anoikis resistance, migration and invasiveness [15, 38-40]. Anoikis-resistant NSCLC cells overexpressing Cav1 exhibit significant increases in anchorage-independent growth, extracellular matrix adhesion, migration, and invasion, whereas Cav1 knockdown by shRNA transfection is able to reverse the metastatic potential [16]. The findings of the present study are consistent with those of our previous study of differential Cav1 expression according to histotype [18], showing that all lung large cell neuroendocrine carcinomas and matched brain metastases were Cav1 negative. In addition, 20% of the primary tumors shifted to Cav1 positivity during metastasis. Many studies, including a recent meta-analysis [17], have confirmed that Cav1 tissue expression is a negative prognostic marker in NSCLC and that it is correlated with differentiation, T stage and lymph node metastasis. However, the clinical significance of Cav1 expression in resected brain metastasis has not yet been investigated. In our study, only Cav1 expression in brain metastasis, and not in the primary tumor, was correlated with poor prognosis and an increased risk of death in the multivariate model, demonstrating its value as an independent prognosticator. Moreover, as age is *per se* an adverse prognostic factor, we investigated the relationship between age and Cav1 expression at the metastatic site, demonstrating that positive Cav1 metastasis was independently associated with a higher risk of death, exclusively in the group of young patients.

A somatic missense mutation in *CAV1* at codon 132, converting proline to leucine (P132L), has been detected in 11-16% of invasive breast cancer patients [41, 42]. As the recombinant expression of the *CAV1* P132L mutant leads to mislocalization and intracellular retention of wild-type *CAV1*, cellular transformation, and promotion of invasive ability [41], a dominant-negative behavior has been suggested for the P132L mutant [41, 43]. Although this mutation was not identified in any of 46 lung cancer cell lines examined by Sunaga *et al*. [15] and has not been described in other studies investigating mutations in lung cancer [38, 44], we detected it in two human adenocarcinoma specimens. Both patients with this *CAV1* P132L mutation had a single brain metastasis, no extracranial tumors and a Karnofsky Performance Status (KPS) of 90. Their survival times (47 and 49 months) were longer than the median survival time of the series (14 months). These findings indicate that mutation of *CAV1* results in a loss of its unfavorable prognostic/predictive value, in contrast with what has been previously described in different oncologic contexts [43].

Predictive role of Cav1 for radiotherapy responsiveness in brain metastasis: In solitary brain metastasis, surgical resection followed by WBRT or stereotactic radiosurgery might be beneficial [2]. However, the adjuvant treatment of these patients still results in disappointing outcomes because WBRT only extends survival by three to six months, even when achieving palliative improvement of neurological symptoms [3]. In our series, radiotherapy was not significantly associated with survival. Nevertheless, among the radiotherapy-treated patients, independent of the intent, Cav1 proved to be a useful marker for predicting radiotherapy responsiveness because the patients with Cav1-negative brain metastasis had a reduced risk of death, whereas those with Cav1 positivity showed an increased risk. These findings are consistent with reports of induction of radiosensitization by Cav-1 knockdown in the same tumor model [10, 11] and radiation-elicited Cav1 expression in pancreatic cancer cell lines [10]. Moreover, low Cav1 expression in pre-treatment biopsies from patients with locally advanced colorectal cancer has been correlated with better rates of local control and overall survival [12].

## CONCLUSIONS

Cav1 expression in brain metastasis from lung cancer is independently predictive of worse outcome and radioresistance. Therefore, in the future development of personalized therapy for advanced oncologic patients, Cav1 could become an additional tool to prognosticate and predict a distinguished subset of patients with brain metastatic lung cancer.

## MATERIALS AND METHODS

### Case collection

Sixty-nine NSCLCs and their matched resected brain metastases were retrieved from consecutive cohorts from the archives of two different institutions, the Pathology Divisions of the University of Torino at Città della Salute e della Scienza (Molinette) Hospital in Turin and San Luigi Hospital in Orbassano, Italy. In 25 cases (36%), brain metastasis was diagnosed synchronously with primary NSCLC, and in three cases (4%), brain metastasis was diagnosed prior to primary lung cancer (1 to 3 months before). In the remaining 41 cases (64%), metastasis was metachronous (mean of 9 months from NSCLC diagnosis, range 1-63). None of the patients had extra-cranial tumors. At the time of neurosurgical resection, the patients with a single brain metastasis comprised 71% of the total patients (49 patients), whereas the remaining 29% of the patients (20 patients) had multiple metastases. The patients with a single brain metastasis underwent conventional surgery. Thirteen out of 69 patients received chemotherapy. Information about brain radiotherapy was available for 49 patients. Among them, 22 patients with a single surgically removed lesion received radiotherapy with an adjuvant intent; conversely, nine patients with multiple brain lesions underwent radiotherapy with a palliative-cytoreductive intent. Whole-brain radiotherapy was administered with the same schedule (30 Gy in 10 fractions) in both patient groups. The group of patients treated with adjuvant radiotherapy had a good clinical performance status (mean KPS of 90) without any significant neurological deficits. The patients treated with cytoreductive whole-brain radiotherapy for active metastatic disease occasionally had some neurological deficits related to the site of the disease, but the KPS did not fall lower than 70, which is mandatory for irradiation. Similarly, all of the patients who did not receive radiotherapy had a KPS of over 70. All of the case data were anonymously recorded. The study received ethical approval from the local Institutional Review Boards.

### Histopathological evaluation

All hematoxylin and eosin (H&E)-stained slides available from surgical specimens were reviewed, and a representative paraffin block was selected for each case. The primary tumor and matched brain metastasis from each patient were independently reviewed by two dedicated pathologists (RS and PC), who confirmed all diagnoses according to the current World Health Organization classification [19].

### Immunohistochemistry

Immunohistochemistry was performed in all cases. Three micrometer-thick serial paraffin sections for each case were processed by immunohistochemistry using an antibody against Cav1 (Santa Cruz, Santa Cruz, CA, USA, rabbit polyclonal, diluted 1/350) with an automated immunostainer (Ventana BenchMark Auto-Stainer, Ventana Medical Systems). A biotin-free, dextran chain-based detection system (EnVysion, Dako, Carpinteria, CA, USA) was used for visualization, and diaminobenzidine (Ventana Medical Systems, Tucson, AZ, USA) was used as a chromogen, according to standard protocols. Vascular endothelium represented an internal positive control.

### Staining interpretation and scoring system

All immunostained slides were analyzed independently by RS, ED and PC, who were blinded to the clinical data. Cav1 staining was assessed as a categorical variable (negative or positive if present in at least 10% of neoplastic cells). In cases of discrepancies, slides were reviewed using a multihead microscope, and a consensus was reached.

### Mutational analysis

Mutational analysis of the Cav1 gene was performed in 54 cases (in both the primary tumor and metastasis) to identify somatic mutations. Genomic DNA extractions were performed with standard methods (NucleoSpin Tissue Kit, Macherey-Nagel, Duren, Germany), and 6 ng of DNA was amplified using specific primers for the human Cav1 gene. Specifically, PCR primers were designed to amplify and sequence exon 2 and exon 3 of Cav1 as follows: Cav-1F-exon2: 5′-GCAGGGACATCTCTACACG-3′; Cav-1R-exon2: 5′-GCCTTGGCTTACCTTGACCA-3′; Cav1F-exon3: 5′-AACCAGAAGGGACACACAG-3′; and Cav-1R-exon3: 5′-AAAGAGTGGGTCACAGACG-3′.

The somatic origin of each mutation was confirmed by the sequencing of both neoplastic and normal DNA obtained by laser-capture microdissection of a non-transformed area adjacent to the neoplastic lesion. For small samples, normal DNA was extracted from a different histological specimen collected from the same patient. Mutations were detected only in malignant tissues. Positive results for mutational analysis were validated at least twice in independent PCRs. Pathologists carrying out these analyses were blinded to the clinical outcomes of the patients.

### Cav-1 fluorescence in situ hybridization (FISH) analysis

FISH was performed using both an alpha satellite probe specific for chromosome 7 (CEP7) directly labeled with green fluorochrome (Cytocell Technologies Ltd., UK) as a control probe and a self-made probe specific for the *CAV1,2* locus, BAC 688K20 (7q31.2). The clone was obtained from Invitrogen (Invitrogen Corporation, USA). UCSC database (http://genome.ucsc.edu July 2008 release) was queried for the localization of the probe. The BAC was expanded, extracted and then directly labeled with SpectrumOrange-dUTP (Abbott Molecular, Europe) using a BioPrime DNA Labeling System (Invitrogen Corporation, USA), according to the manufacturer's instructions. To analyze the position and strength of the signal, the presence/absence of background and cross-hybridization, and the hybridization efficiency, the BAC clone was tested using metaphase and interphase cells from healthy donors obtained using conventional cytogenetic methods. FISH was routinely performed with the two probes CAV1,2 and CEP7 on formalin-fixed, paraffin-embedded tissue. Images of red (CAV1,2) and green (CEP7) spots in areas with significant signal were automatically acquired using 13 focus planes, with Metafer software by a MetaSystem scanning station (Carl Zeiss MetaSystems GmbH, Altlussheim, Germany) equipped with an AxioImager epifluorescence microscope. The automatically acquired images were analyzed with Isis software (Zeiss). Eighty to 100 non-overlapping neoplastic nuclei with two control green signals (2G) were analyzed for each case and were scored as follows: 2R, normal nuclei; R > 3, CAV gain; R > 5, CAV amplification; and R < 2, CAV loss.

### Statistical analysis

Correlations between Cav1 expression and the clinico-pathological variables were quantified using the chi-square test or Fisher's exact test, as appropriate. Kaplan-Meier curves and the log rank test were used to assess differences in survival between the groups of patients. Overall survival was defined as the time elapsed from the date of lung cancer diagnosis to the date of death or the last visit. Hazard ratios (HRs) and 95% confidence intervals (CIs) were estimated by the Cox proportional hazards model for multivariate survival analysis. The log-likelihood ratio test based on a chi-square approximation was used to perform model selection as appropriate. Statistical significance was set at a level of 0.05. All analyses were conducted using version 2.12.1 of R statistical package (www.r-project.org).
